# Body Mass Index and Prognosis of COVID-19 Infection. A Systematic Review

**DOI:** 10.3389/fendo.2020.00562

**Published:** 2020-08-14

**Authors:** Karina Colombera Peres, Rachel Riera, Ana Luiza Cabrera Martimbianco, Laura Sterian Ward, Lucas Leite Cunha

**Affiliations:** ^1^Laboratory of Cancer Molecular Genetics, School of Medical Sciences, University of Campinas, Campinas, Brazil; ^2^Discipline of Evidence-Based Medicine, Escola Paulista de Medicina, Universidade Federal de São Paulo, São Paulo, Brazil; ^3^Centre of Health Technology Assessment, Hospital Sírio-Libanês, São Paulo, Brazil; ^4^Postgraduate Program in Health and Environment, Universidade Metropolitana de Santos, São Paulo, Brazil; ^5^Centro Universitário São Camilo, São Paulo, Brazil; ^6^Department of Medicine, Escola Paulista de Medicina, Universidade Federal de São Paulo, São Paulo, Brazil

**Keywords:** obesity, body mass index, SARS-CoV-2, COVID-19, risk factor

## Abstract

A better understanding of the SARS-CoV-2 virus behavior and possible risk factors implicated in poor outcome has become an urgent need. We performed a systematic review in order to investigate a possible association between body weight and prognosis among patients diagnosed with COVID-19. We searched in Cochrane Library, EMBASE, MEDLINE, WHO-Global Literature on Coronavirus Disease, OpenGrey, and Medrxiv. We used the ROBINS-I tool or Cross-Sectional/Prevalence Study Quality tool from AHRQ, to evaluate the methodological quality of included studies. Nine studies (two prospective cohorts, four retrospective cohorts and three cross-sectional) were included and assessed the relationship between obesity and COVID-19 prognosis. Risk of bias of the included studies ranged from moderate to critical. Clinical and methodological heterogeneity among them precluded meta-analyses. Most of the included studies showed some degree of association to: (a) higher BMI and worse clinical presentation and (b) obesity and need of hospitalization. The results were inconsistent about the impact of obesity on mortality. Based on limited methodological quality studies, obesity seems to predict poor clinical evolution in patients with COVID-19. Further studies with appropriate prospective design are needed to reduce the uncertainty on this evidence.

## Introduction

A better understanding of the SARS-CoV-2 virus behavior has become an urgent need as the pandemic caused continues to plague the world adding more and more victims. A series of reports have looked for risk factors in order to provide means of prevention and treatment to the population. The first Chinese publications made clear that age may impact prognosis (1–3), but with the advance of COVID-19 to western European and North American countries, some novel factors have emerged as determinants of risk and poor outcome. In contrast to China, there is a high prevalence of obesity in these countries (4) that may help explain, at least in part, the reason why obesity has just emerged as a marker of unfavorable clinical evolution.

The prevalence of obesity has rapidly increased over the years (5), especially among elderly (6). Obesity is a multifactorial disorder characterized by excessive fat accumulation and an increment of proinflammatory cytokines, which entails a constant state of immune deregulation (7). This chronic deregulation may interfere with immune homeostasis and impair the effectiveness of the immune response. It is not without reason that obesity has been implicated in poor outcomes among many clinical conditions and high all-cause mortality (8).

However, although it accumulates rapidly, data on the role of obesity on COVID-19 risk and prognosis are still confusing and hard to interpret. Scrambling to learn more about the virus, doctors and scientists try to rapidly share their findings generating a large flood of publications that has put new strain on a scientific process accustomed to vetting and publishing new results much more slowly.

Herein, we perform a systematic review in order to evaluate if overweight and obesity may predict poor outcome in patients with COVID-19.

## Objectives

To investigate a possible association between body weight and prognosis among patients diagnosed with COVID-19.

The clinical question is, as structured through the PECO acronym: (P, population): individuals with COVID-19; (E, exposure): overweight or obesity); (C, comparator): normal body weight; (O, outcomes): clinical, laboratory and image outcomes on COVID-19.

## Methods

### Study Design and Setting

This was a systematic review carried out in the Universidade Federal de São Paulo (Unifesp) through a collaboration with the University of Campinas (Unicamp), Brazil. The study was conducted in accordance with the AMSTAR-2 (Assessing the Methodological quality of Systematic Reviews) (9). The protocol was prospectively registered at the PROSPERO database (registration number CDR42020182189, https://www.crd.york.ac.uk/prospero/display_record.php?RecordID=182189). This reporting was written following the PRISMA statement (10).

### Criteria for Including Studies

#### Types of Studies

We considered any study design using a comparative group as follows: controlled trials (randomized, quasi-randomized, or non-randomized) that conducted subgroup analyses according to body weight, cohort and case-control studies, and analytic cross-sectional studies with a control group.

#### Types of Participants

Adults or children with confirmed diagnosis of COVID-19, in accordance with World Health Organization criteria (11).

#### Types of Exposure

We considered any definition of overweight or obesity, as assumed by the authors of primary studies. However, only similar definitions were evaluated together into quantitative or qualitative synthesis.

### Outcomes

We considered all clinical, laboratory and image outcomes as presented by the authors of primary studies. However, we prioritized the outcomes below:

Primary outcomes:

- All-cause mortality;
- Serious adverse events: assessed by the rate of participants who experienced at least one serious adverse event, as per defined as those that are life-threatening; which may lead to death, requirement of a treatment in an emergency room, hospitalization (initial or prolonged), disability or permanent damage, or congenital anomaly/birth defect (12).
- SARS-CoV-2 acute respiratory syndrome: assessed by the rate of participants who progressed to acute respiratory syndrome.
- Clinical status, assessed by the Ordinal Scale for Clinical Improvement—World Health Organization (scale from 0 to 7, the higher the score, the worse the clinical condition), as defined by the World Health Organization (WHO) (13).

Secondary outcomes:

- Mortality related to SARS-CoV-2 infection (COVID-19);
- Any adverse event: assessed by the rate of participants who experienced at least one adverse event.
- Time to clinical improvement, defined as a reduction of at least two points in the score of the Ordinal Scale for Clinical Improvement—World Health Organization (scale from 0 to 7, the higher the score, the worse the clinical condition), as defined by the World Health Organization (WHO) (13).
- Hospitalization in an intensive care unit;
- Need for invasive mechanical ventilation;
- Length of hospitalization.
- Length of hospitalization in intensive care unit;
- Length of invasive mechanical ventilation;
- Rate of negative PCR viral load (any specimen).

We assessed all dichotomous outcomes listed above at any time point. However, we only pooled similar time points together: short term (up to 1 month, inclusive) or long term (more than 1 month). When a study reported an outcome more than once in the same period, we considered the last measurement.

### Search for Studies

A comprehensive search of the literature was carried out using an electronic search with no restriction regarding date, language or status of publication. Sensitive search strategies (Supplementary File 1) were developed for the following databases:

- Cochrane Library (via Wiley);
- EMBASE (via Elsevier);
- MEDLINE (via PubMed);
- World Health Organization—Global Literature on Coronavirus Disease (https://search.bvsalud.org/global-literature-on-novel-coronavirus-2019-ncov/).

A search for gray literature was conducted in the Opengrey database (https://opengrey.eu) and for preprint studies in the Medrxiv (https://www.medrxiv.org/). Manual search was performed in the reference lists of the relevant studies.

### Selection of Studies

The selection process was conducted in a two-stage process aided by the Rayyan platform (14). In the first phase, two review authors independently assessed all titles and abstracts retrieved by the search strategies. Studies marked as “potentially eligible” were then screened at the second phase, which consisted in the reading of the full text to confirm its eligibility. Any divergence was solved by a third reviewer. Studies excluded in the second phase were presented in the “excluded studies table” and the reasons for exclusion as well.

### Data Extraction

The procedures for data extraction were performed by two independent reviewers and a pre-established data extraction form was used. Disagreements in this process were solved by a third reviewer.

### Methodological Quality of Studies

The methodological quality of the included studies was evaluated by two independent reviewers by the use of validated tools for each study design, as following:

- Randomized controlled trial: Cochrane Risk of Bias Table (15);
- Non-randomized, quasi-randomized trial: ROBINS-I (16);
- Cohort or case-control: ROBINS-I (16). ROBINS-I was used as there are, as yet, no draft versions of ROBINS-E available. The domains “classification of interventions” and “deviations from intended interventions” were adapted to consider “exposures” instead of “interventions.”
- Cross-sectional: Cross-Sectional/Prevalence Study Quality, Agency for Healthcare Research and Quality (17).

### Unity of Analysis and Missing Data

The unit of analysis was the individual. Considering the context requiring a rapid answer, the authors from primary studies were not contacted for missing data.

### Data Analysis and Presentation

Depending on data availability and homogeneity of studies, we planned to pool results from similar studies by random-effects meta-analyses (software Review Manager 5.3). Risk ratios (or odds ratios) and mean differences would be calculated for dichotomous and continuous data, respectively. A 95% confidence interval would be considered for the analyses. When meta-analysis was not possible the results were presented as qualitative synthesis (descriptive presentation).

### Heterogeneity Assessment

Methodological and clinical diversity of included studies would be considered for conducting or not meta-analyses. The existence of statistical heterogeneity would be evaluated by Chi^2^ test and its extension by the *I*^2^ test (*I*^2^ ≥ 50% indicates high heterogeneity among studies).

### Additional Analyses

We planned to conduct the following subgroup analyses: (a) presence of diabetes and/or hypertension and (b) age of participants (< 65 vs. ≥65).

We planned to conduct the following subgroup analyses: (a) fixed effects vs. random effects model meta-analysis. When the results of fixed effect meta-analysis provide a different result, both would be reported; (b) excluding from analysis studies at high risk of bias; and (c) excluding from analysis unpublished studies or those available exclusively in a pre-print version and not peer reviewed.

Investigation of publication bias assessment was planned by visual inspection of funnel plots for meta-analysis with at least 10 studies.

However, due to heterogeneity between included studies it was not possible to conduct meta- analyses nor additional analyses.

## Results

### Results From Search

**Figure 1:**
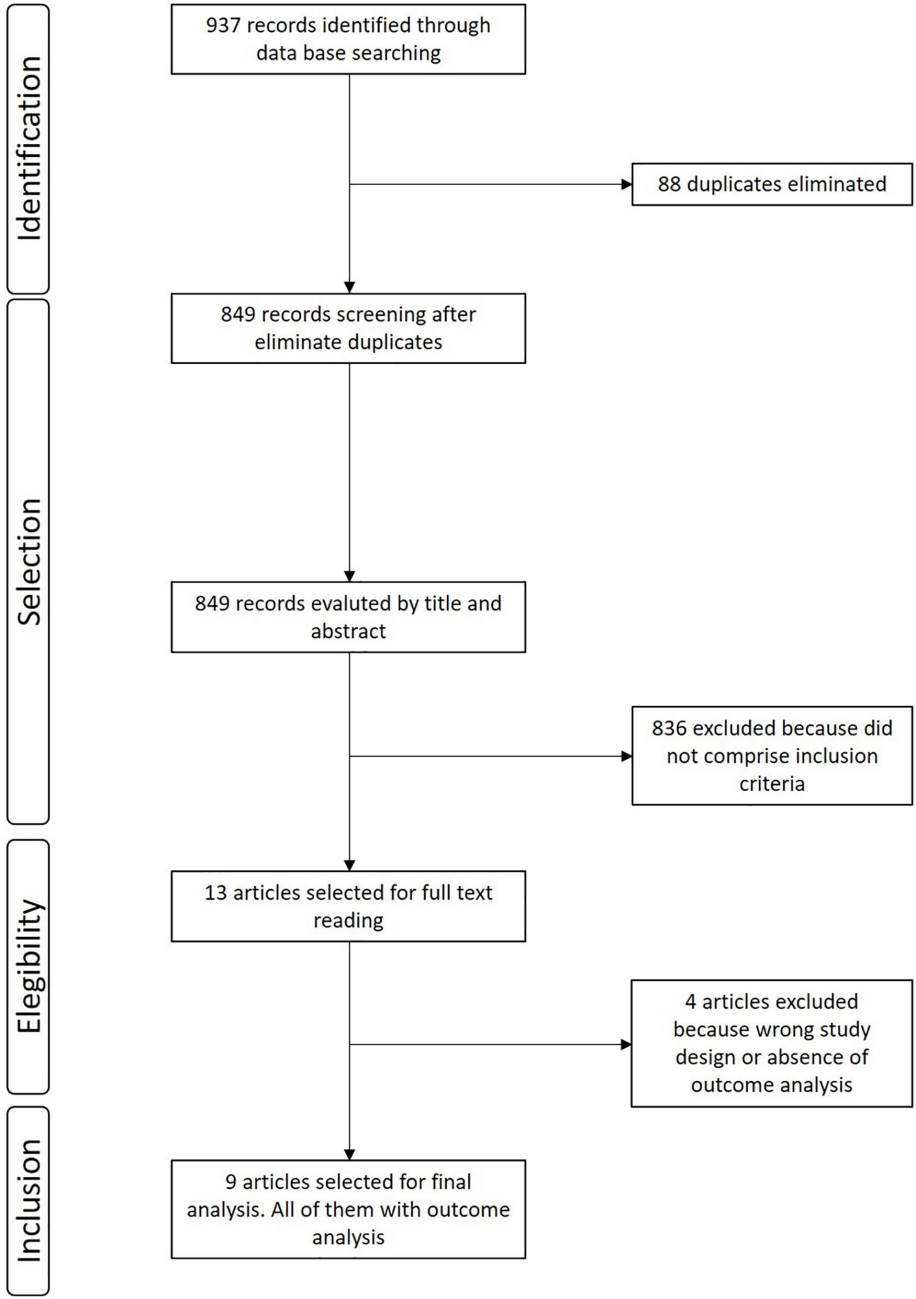
Flow diagram of the selection process.

The search retrieved 937 records. After excluding 88 duplicates, we screened the titles and abstracts of 849 references, excluded 836 that did not comprise inclusion criteria, and selected 13 for full text reading. We excluded four studies (detailed below). Therefore, the review included nine observational studies. The flow diagram of the process of study identification and selection is presented in Figure 1.

### Results From Included Studies

This systematic review included nine studies: two prospective cohort studies (18, 19), four retrospective cohort studies (20–23), and three cross-sectional studies (24–26). Table 1 presents the main characteristics of the included studies. Table 2 summarizes the studies excluded after selection.

**Table 1:** Main characteristics of the included studies.

**References**	**Study design**	**Number of patients with COVID-19**	**Exposure**	**Outcomes**	**Main results**
Argenziano et al. (24)	Cross-sectional	1000	Mean BMI kg/m^2^	Level of hospital care	Mean BMI of admitted ICU patients was significantly higher than BMI of admitted patients in all other levels of care (31.2 ± 8.0 vs. 29.9 ±7.24 kg/m^2^) (DM 1.30, 95% CI 95% 0.15–2.45; *p* = 0.03)
Bello-Chavolla et al. (25)	Cross-sectional	8261	BMI > 30 kg/m^2^ Age > 65 years	Mortality Hospitalization Pneumonia ICU admission Invasive mechanical ventilation	Compared to non-obese, obese patients had a significantly increased risk of: • Mortality (13.6 vs. 7.1%, *p* < 0.001; HR 7.56 95%CI 5.79–9.87) • Hospitalization (47.3 vs. 34.4%, *p* < 0.001) • Pneumonia (36.4 vs. 25.9%, *p* < 0.001) • ICU admission (7.2 vs. 4.2%, *p* = 0.034) • Invasive ventilation (6.9 vs. 4%, *p* = 0.029)
Cummings et al. (18)	Prospective cohort	257	Severe obesity (BMI ≥ 35 kg/m^2^)	Rate of in-hospital death	No difference between obese and non-obese patients in mortality (HR 0.94, 95% CI 0.55–1.77)
Lighter et al. (20)	Retrospective cohort	3,615	BMI < 30 vs. BMI 30–34 and BMI ≥ 35 kg/m^2^	Age (>60 and < 60 years) Hospital admission	Age > 60 years: Compared to non-obese (BMI < 30), there was no difference between groups in: • Admission to acute care: BMI 30–34: RR 0.9, 95% CI 0.6–1.2, *p* = 0.39 BMI ≥35: RR 0.9, 95% CI 0.6–1.3, *p* = 0.59 • Admission to ICU: BMI 30–34: RR 1.1, 95% CI 0.8−1.7, *p* = 0.57 BMI ≥35: RR 1.5, 95% CI 0.9–2.3, *p* = 0.10 Age < 60 years: Compared to non-obese (BMI < 30), obese patients had more: • Admission to acute care: BMI 30–34: RR 2.0, 95% CI 1.6–2.6, *p* < 0.0001 BMI ≥35: RR 2.2, 95% CI 1.7–2.9, *p* < 0.0001 • Admission to ICU: BMI 30–34: RR 1.8, 95% CI 1.2–2.7, *p* = 0.006 BMI ≥35: RR 3.6, 95% CI 2.5–5.3, *p* < 0.0001.
Liu et al. (21)	Retrospective cohort	30	BMI (mean, SD)	COVID-19 severity (mild vs. severe)	Severe COVID patients had a significantly higher mean BMI (27.0 ± 2.5) than mild patients (22.0 ± 1.3) (*p* < 0.001).
Peng et al. (22)	Retrospective cohort	112	BMI ≥ 25 (obese plus overweight) vs. BMI < 24 kg/m^2^ (eutrophic or lean)	Mortality	Obese patients had a significant increased risk of mortality comparing to non-obese (18.92 vs. 88.24%, *p* < 0.001); Mean BMI of the critical group (ICU need) was significantly higher than the general group (*n* = 16 vs. 96; *p* = 0.003).
Petrilli et al. (26)	Cross-sectional	4,103	BMI < 30 vs. BMI 30–40 and BMI >40 kg/m^2^	Hospitalization	•Non-hospitalized group: BMI 30–40: 12.2% (256 patients) BMI >40: 2.3% (48 patients) • Hospitalized group: BMI 30–40: 33.0% (659 patients) BMI >40: 6.9% (137 patients) BMI >40 was significantly associated with hospitalization when compared to BMI < 30 (OR 6.2, 95% CI 4.2–9.3)
Simonnet et al. (23)	Retrospective cohort	124	BMI categories: 18.5 to < 25; 25 to < 30; 30 to < 3; ≥35 kg/m^2^	Invasive mechanical ventilation	Obese patients (BMI ≥ 35) had a significant increased risk of invasive ventilation need, comparing to non-obese (BMI < 25) (OR 7.36, 95% CI 1.63–33.14, *p* = 0.021)
Zheng et al. (19)	Prospective cohort	66 (with metabolic associated fatty liver disease)	BMI > 25 kg/m^2^	COVID-19 severity	Severe patients had a significantly higher proportion of obese than non-severe (89.5 vs. 59.6%, *p* = 0.021). Obese patients with metabolic associated fatty liver diseases had a significantly increased risk of severe COVID-19 (OR 6.32, 95% CI 1.16–34.54, *p* = 0.033)

*n, number of participants; BMI, body mass index; ICU, Intensive care unit; SD, Standard deviation; HR, Hazard ratio*.

**Table 2:** Studies excluded after selection.

**References**	**Reason for exclusion**
Malavazos et al. (27)	Different study design (narrative review).
Ryan and Caplice (28)	Different study design (narrative review).
Garg et al. (29)	Only data of obesity prevalence, with no outcome association analysis.
Richardson et al. (30)	Only data of obesity prevalence, with no outcome association analysis.

BMI was the only measure used as a criteria for classifying body weight, considered as a continuous or ordinal scale variable. The included studies had different study designs and considered different outcomes of interest.

Due to these clinical and methodological heterogeneity among included studies it was not appropriate to conduct meta-analyses.

### Methodological Assessment of Studies

Risk of bias assessment of the included studies and reasons for judgement are presented in Tables 3, 4. Overall, cohort studies were classified as critical to moderate risk of bias, and cross-sectional studies varied between 36 and 63% of agreement with bias domains.

**Table 3:** Risk of bias of cohort studies: ROBINS-I (16).

**Study/Bias domain**	**Confounding**	**Selection of participants**	**Classification of interventions**	**Deviations from intended interventions**	**Missing data**	**Measurement of the outcome**	**Selection of the reported result**	**Overall**
Cummings et al. (18)	Critical risk of bias It is likely that one or more prognostic variables are present unbalanced among the compared groups	Moderate risk of bias Prospective study; start of follow-up coincide for most participants	Moderate risk of bias Criteria used to define the exposure was described	Moderate risk of bias Probably no deviation happened	Low risk of bias Data from cohort were apparently complete	Low risk of bias Objective outcome assessed (mortality) could not be influenced by outcome assessors	Critical risk of bias Participants selected from a larger group and it is not possible to exclude bias related to the reporting of outcomes	Moderate risk of bias
Lighter et al. (20)	Critical risk of bias It is likely that one or more prognostic variables are present unbalanced among the compared groups	Critical risk of bias Retrospective study	Moderate risk of bias Criteria used to define the exposure was described	Moderate risk of bias Probably no deviation happened	No information No information on which to base a judgement on losses during the study period	Critical risk of bias It is very likely that the subjective outcomes assessed were influenced by knowledge of the prognostic factor	Critical risk of bias Participants selected from a larger group and it is not possible to exclude bias related to the reporting of outcomes	Critical risk of bias
Liu et al. (21)	Critical risk of bias It is likely that one or more prognostic variables are present unbalanced among the compared groups	Critical risk of bias Retrospective study	Moderate risk of bias Criteria used to define the exposure was described	Moderate risk of bias Probably no deviation happened	No information No information on which to base a judgement on losses during the study period	Critical risk of bias It is very likely that the subjective outcomes assessed were influenced by knowledge of the prognostic factor	Critical risk of bias Participants selected from a larger group and it is not possible to exclude bias related to the reporting of outcomes	Critical risk of bias
Peng et al. (22)	Critical risk of bias It is likely that one or more prognostic variables are present unbalanced among the compared groups	Critical risk of bias Retrospective study	Moderate risk of bias Criteria used to define the exposure was described	Moderate risk of bias Probably no deviation happened	Low risk of bias Data from cohort were apparently complete	Critical risk of bias It is very likely that the subjective outcomes assessed were influenced by knowledge of the prognostic factor	Critical risk of bias Participants selected from a larger group and it is not possible to exclude bias related to the reporting of outcomes	Critical risk of bias
Simonnet et al. (23)	Critical risk of bias It is likely that one or more prognostic variables are present unbalanced among the compared groups	Critical risk of bias Retrospective study	Moderate risk of bias Criteria used to define the exposure was described	Moderate risk of bias Probably no deviation happened	Low risk of bias Data from cohort were apparently complete	Critical risk of bias It is very likely that the subjective outcomes assessed were influenced by knowledge of the prognostic factor	Low risk of bias All patients admitted to intensive care for SARS-CoV-2 were analyzed	Critical risk of bias
Zheng et al. (19)	Critical risk of bias It is likely that one or more prognostic variables are present unbalanced among the compared groups	Moderate risk of bias Prospective study; start of follow-up coincide for most participants	Moderate risk of bias Criteria used to define the exposure was described	Moderate risk of bias Probably no deviation happened	Low risk of bias Data from cohort were apparently complete	Critical risk of bias It is very likely that the subjective outcomes assessed were influenced by knowledge of the prognostic factor	Low risk of bias All patients with COVID-19 and with metabolic associated fatty liver disease were analyzed	Moderate risk of bias

*Low risk of bias: The study is comparable to a well-performed randomized trial with regard to this domain*.

*Moderate risk of bias: The study is sound for a non-randomized study with regard to this domain but cannot be considered comparable to a well-performed randomized trial*.

*Serious risk of bias: The study has some important problems in this domain*.

*Critical risk of bias: The study is too problematic in this domain to provide any useful evidence on the effects of intervention*.

*No information: No information on which to base a judgement about risk of bias for this domain*.

**Table 4:** Risk of bias of cross-sectional studies: Cross-Sectional/Prevalence Study Quality, Agency for Healthcare Research and Quality (AHRQ) (17).

**Domain**	**Argenziano et al. (24)**	**Bello-Chavolla et al. (25)**	**Petrilli et al. (26)**
1 Define source of information (survey, record review)	Y	Y	Y
2 List inclusion and exclusion criteria for exposed and unexposed subjects (cases and controls) or refer to previous publications	Y	Y	Y
3 Indicate time period used for identifying patients	Y	N	Y
4 Indicate whether or not subjects were consecutive if not population-based	Y	N	Y
5 Indicate if evaluators of subjective components of study were masked to other aspects of the status of the participants	Y	N	Y
6 Describe any assessments undertaken for quality assurance purposes (e.g., test/retest of primary outcome measurements)	N	N	N
7 Explain any patient exclusions from analysis	U	Y	N
8 Describe how confounding was assessed and/or controlled	N	Y	Y
9 If applicable, explain how missing data were handled in the analysis	NA	U	Y
10 Summarize patient response rates and completeness of data collection	U	N	U
11 Clarify what follow-up, if any, was expected and the percentage of patients for which incomplete data or follow-up was obtained	N	N	N
Number (percentage) of domain agreement	5/10 (50%)	4/11 (36%)	7/11 (63%)

*Y, Yes; N, No; U, Unclear; NA, Not applicable*.

## Discussion

We reviewed data from 17,568 patients with SARS-CoV-2 infection, included in nine studies. Most of these studies highlighted some level of association between obesity and disease severity, encompassing hospitalization rate, admission to ICU, invasive ventilation need and mortality. According to validated tools, these studies presented moderate to critical risk of bias, which limits the reliability in the results.

According to COVIDView database of Centers for Disease Control and Prevention (CDC) in the USA, until May 2, 2020 the overall rate for COVID-19-associated hospitalization were 162.2 per 100,00 in individuals 65 years and older, decreasing to 79.0–26.2 for individuals < 65 years. Furthermore, preliminary data showed that about 91.5% of hospitalized patients present at least one underlying medical condition. Besides obesity, the most common critical comorbidities observed in the hospitalized COVID-19 patients were hypertension, metabolic disease, cardiovascular and pulmonary diseases (31).

Three of 4 North American studies showed increased BMI among patients who required hospitalization. Argenziano et al. (24) also described that patients who require in-hospital admission had more chronic diseases, such as hypertension, diabetes, and obesity. To date, ICU patients presented significantly higher BMI compared to those admitted in the emergency or inpatient floors. Lighter and colleagues (20) analyzed retrospectively a cohort of 3,615 patients positive for COVID-19 stratified by age. Thirty-eight percent (38%) of these patients presented BMI >30 kg/m^2^. An increased risk of hospitalization in acute care or ICU was demonstrated for patients < 60 years older with obesity (BMI 30–34 kg/m^2^) and severe obesity (BMI ≥35 kg/m^2^) compared to patients BMI < 30 kg/m^2^. Once younger patients generally do not represent higher risk for a severe presentation of COVID-19, authors suggest that obesity may be an unrecognized risk factor for hospital care. In a cross-sectional study, Petrilli et al. (26) showed that hospitalized patients were more likely to be male and present cardiovascular diseases, diabetes and obesity. In fact, as confirmed by a multivariate analysis, obesity (BMI > 40 kg/m^2^), older age (≥65 years) and history of heart failure were independent predictors of unfavorable outcome. Cummings et al. (18) observed similar prevalence of obesity among hospitalized patients. However, authors failed to demonstrate that obesity is a predictor of mortality.

Two cohort studies evaluate the severity of COVID-19 disease in Chinese patients. The retrospective study by Liu et al. (21) evaluated 30 medical staff infected with novel coronavirus in January, 2020. Most of them presented a common type of the disease (*n* = 26) and four patients a more severe condition defined as pulmonary insufficiency. Until the end of the study, 80% of the patients were discharged, none of them needed critical hospital care or died. In relation to obesity the authors reported higher BMI in patients with severe compared to the mild presentation. Zheng et al. (19) prospectively evaluated 66 patients with metabolic associated fatty liver disease (MAFLD) stratified by obesity status dividing patients according to severe and non-severe COVID-19 based on the National Health Commission & State Administration of Traditional Chinese Medicine. Frequency of obesity was higher between severe disease patients compared to non-severe, furthermore MAFLD patients with concurrent obesity had more severe presentation of the disease. Indeed, obesity in patients with MAFLD increased the risk of severe illness in almost 6-fold (unadjusted OR 5.77, 95% CI 1.19–27.91, *p* = 0.029). After adjustment for age, sex, smoking, diabetes, hypertension, and dyslipidemia, association with obesity and COVID-19 remained significant and confirmed obesity as an independent marker of critical illness. However, as commented by Hussain et al. (32), MAFLD and obesity are rarely considered as independent conditions, in the cases of concurrent diseases they coexist due to obesity. A third Chinese study (22) demonstrated that mean BMI of the 16 patients who needed ICU care (25.5 kg/m^2^) were higher than the general group (22.0 kg/m^2^). Between the 17 deaths reported, 88% had BMI >25 kg/m^2^. Most of the deceased patients also presented hypertension, coronary heart disease and heart insufficiency.

In a Mexican study (25), obese patients, as expected, had higher proportions of other comorbidities as hypertension, diabetes, cardiovascular disease, asthma, and chronic obstructive pulmonary disease (COPD). Increased lethality of COVID-19 was reported specially in patients with diabetes, early onset diabetes (< 40 years), concurrent obesity or several concurrent comorbidities (*p* < 0.001). As presented in Table 1, obese patients had higher risks of hospitalization, pneumonia, ICU admission, invasive ventilation and 7-fold increased risk of mortality.

Simonnet et al. (23) described that the distribution of BMI categories in COVID-19 patients admitted to ICU care in France differed from the control patients with non-SARS-Cov-2 respiratory disease. The frequency of obesity (BMI > 30 kg/m^2^, 47.6%) and severe obesity (BMI > 35 kg/m^2^, 28.2%) were higher among patients with COVID-19 infection compared to control patients (25.2 and 10.8%, respectively). Besides, the median of BMI (31.1 kg/m^2^) of the 85 patients who required invasive ventilation was higher than the patients who did not (27.0 kg/m^2^, *n* = 39). An univariate logistic regression analysis showed that BMI ≥35 kg/m^2^ (vs. BMI < 25 kg/m^2^) was a risk for need of invasive ventilation (OR 6.75, 95%CI 1.76–25.85, *p* = 0.015), remaining significant after adjustment for age, diabetes and hypertension in a multivariate analysis (OR 7.36 95% CI 1.63–33.14, *p* = 0.021).

This systematic review presents some strengths including the use of stringent methods of Cochrane reviews, reproduced in a short term due to the need of rapid responses to guide clinical decisions during the pandemic. The search for studies was highly sensitive and it was conducted in formal databases, preprint, and gray literature repositories and specific sources for COVID-19 as well.

The present study has some limitations. The included studies adopted different methods to assess obesity as a predictor of poor outcome precluding a meta-analysis. Once COVID-19 is a public health emergency, a considerable amount of research is being published every week and it is possible that recent articles may not be included in the final version of our review. For assessing the risk of bias of cohort studies, we slightly adapted the ROBINS-I tool, since there are, as yet, no draft versions of ROBINS-E available. This adaptation was inconspicuous and sought to preserve the definition of domains.

In summary, our systematic review suggests that obesity is likely to be a predictor of poor outcome in patients with COVID-19, in all continents. Obesity is associated with several clinical conditions (e.g., diabetes and hypertension). It is associated with restrictive lung ventilatory defect, which may worsen the severe respiratory failure syndrome. In obesity, the dysfunctional adipocytes produce massive amounts of pro-inflammatory cytokines, which entails a chronic inflammation, harming innate and adaptative immune responses (33–35). The increase in pro-inflammatory cytokines observed among obese patients may add to the inflammatory response triggered by the SARS-CoV-2, and both contribute to poor outcome and high all-cause mortality (7, 8). Likewise, obesity is a well-established risk factor for cardiovascular disease, which triggers diverse physiologic alterations that include activation of renin-angiotensin-aldosterone system, reduction of vasculo-protective effects, upregulation of procoagulant factors, downregulation of anticoagulant factors and chronic oxidative stress and inflammation (36–38). Hence, obese patients with COVID-19 may benefit from an aggressive approach, including eager evaluation and early hospitalization. In addition, health politics may assure obese patients prompt access to the health care system. The investigation of the mechanisms that may be underlying the association between obesity and poor outcome in patients with COVID-19 will certainly help the understanding of this subject. Therefore, further studies with appropriate prospective design are needed to reduce the uncertainty on this evidence.

## Data Availability Statement

All datasets presented in this study are included in the article/Supplementary Material.

## Author Contributions

KP contributed to the design, critical review of the literature and data, composition of the manuscript, and final approval. RR and AM contributed to the design, critical review of the literature and data, risk of bias, composition of the manuscript, and final approval. LW contributed to the design, selection of the notable articles for review, critical review of the literature and data, composition of the manuscript, clinical and translational orientation, and final approval. LC contributed to the conception and design, selection of the notable articles for review, critical review of the literature and data, composition of the manuscript, and final approval. All authors contributed to the article and approved the submitted version.

## Conflict of Interest

The authors declare that the research was conducted in the absence of any commercial or financial relationships that could be construed as a potential conflict of interest.
