# Supplementary material for: Body Mass Index and Prognosis of COVID-19 Infection. A Systematic Review
Source: Front Endocrinol (Lausanne). 2020 Aug 14;11:562. doi: 10.3389/fendo.2020.00562 (PMC7456965; doi:10.3389/fendo.2020.00562)
Supplement: Supplementary file 1 [file Table_1.DOCX]

Supplementary Material

# **Supplementary file 1.** Strategies for electronic searches (23 April 2020)

| **Database** | **Search strategy** | **Results** |
| --- | --- | --- |
| Cochrane Library  23 April 2020 | #1 MeSH descriptor: [Coronavirus] explode all trees  #2 "COVID-19" OR (COVID) OR (Coronavirus) OR (SARS-CoV-2) OR (Coronaviruses) OR (Deltacoronavirus) OR (Deltacoronaviruses) OR "Munia coronavirus HKU13" OR (Coronavirus HKU15) OR (Coronavirus, Rabbit) OR (Rabbit Coronavirus) OR (Coronaviruses, Rabbit) OR (Rabbit Coronaviruses) OR "Bulbul coronavirus HKU11" OR "Thrush coronavirus HKU12"  #3 #1 OR #2 | 145 |
| Embase | #1 exp SARS coronavirus/  #2 exp coronaviridae/ or coronaviridae infection/ or coronavirinae/ or coronavirus infection/  #3 #1 or #2  #4 exp obese patient/ or exp obesity/ or exp body weight disorder/ or exp overnutrition/ or exp abdominal obesity/ or exp adolescent obesity/ or exp childhood obesity/ or exp diabetic obesity/ or exp experimental obesity/ or exp lipedema/ or exp maternal obesity/ or exp metabolic syndrome x/ or exp metabolically benign obesity/ or exp morbid obesity/ or exp obesity hypoventilation syndrome/ or exp sarcopenic obesity/ or exp obesity management/  #5 exp overnutrition/ or exp nutritional disorder/ or exp hyperalimentation/ or exp hypervitaminosis/ or exp obesity/  #6 exp body mass/ or exp "weight, mass and size"/  #7 exp morbid obesity/  #8 #4 or #5 or #6 or #7  #9 #3 and# 8 | 385 |
| MedRxiv / bioRxiv 24 April 2020 | #1 Obesity AND COVID  #2 Overweight AND COVID  #3 BMI AND COVID  #4 #1 OR #2 OR #3 | 122 |
| MEDLINE (via PubMed)  23 April 2020 | #1 "Coronavirus"[Mesh] OR "COVID-19" OR (COVID) OR (Coronavirus) OR (SARS-CoV-2) OR (Coronaviruses) OR (Deltacoronavirus) OR (Deltacoronaviruses) OR "Munia coronavirus HKU13" OR (Coronavirus HKU15) OR (Coronavirus, Rabbit) OR (Rabbit Coronavirus) OR (Coronaviruses, Rabbit) OR (Rabbit Coronaviruses) OR "Bulbul coronavirus HKU11" OR "Thrush coronavirus HKU12"  #2 "Obesity"[Mesh] OR Obesity  #3 "Body Weight"[Mesh] OR "Body Weight" OR “Body Weights” OR “Weight, Body” OR “Weights, Body”  #4 "Obesity, Abdominal"[Mesh] OR "Obesity, Abdominal" OR “Abdominal Obesities” OR “Obesities, Abdominal” OR “Abdominal Obesity” OR “Central Obesity” OR “Central Obesities” OR “Obesities, Central” OR “Obesity, Central” OR “Obesity, Visceral” OR “Visceral Obesity” OR “Obesities, Visceral” OR “Visceral Obesities”  #5 "Abdominal Fat"[Mesh] OR "Abdominal Fat" OR “Abdominal Fats” OR “Fats, Abdominal” OR “Fat, Abdominal” OR “Abdominal Adipose Tissue” OR “Adipose Tissue, Abdominal”  #6 "Overweight"[Mesh] OR Overweight  #7 "Adiposity"[Mesh] OR Adiposity  #8 "Overnutrition"[Mesh] OR Overnutrition OR Hypernutrition  #9 "Body Mass Index"[Mesh] OR "Body Mass Index"OR “Index, Body Mass” OR “Quetelet Index” OR “Index, Quetelet” OR “Quetelet's Index” OR “Quetelets Index”  #10 "Obesity, Morbid"[Mesh] OR "Morbid Obesities" OR "Obesities, Morbid" OR "Obesity, Severe" OR "Obesities, Severe" OR "Severe Obesities" OR "Severe Obesity" OR "Morbid Obesity"  #11 #2 OR #3 OR #4 OR #5 OR #6 OR #6 OR #7 OR #8 OR #9 OR #10  #12 #1 AND #11 | 176 |
| Opengrey  24 April 2020 | #1 "COVID-19" OR (COVID) OR (Coronavirus) OR “SARS-CoV-2” OR (Coronaviruses) OR (Deltacoronavirus) OR (Deltacoronaviruses) OR “SARS CoV 2” | 75 |
| WHO - Global Literature on Coronavirus Disease | #1 obesity OR overweight OR BMI | 34 |
| Total | ------ | 937 |
